# LAR, FAR, and PLR as prognostic factors in high-grade urothelial carcinoma of the bladder after surgery

**DOI:** 10.3389/fonc.2025.1566848

**Published:** 2025-03-11

**Authors:** Huadong Xie, Yuanbi Huang, Chengjie Ban, Wei Wei, Han Tang, Qingming Huang, Zhengwei Su, Zhi Cheng, Tianling Liao, Kangji Liao, Liquan Zhou, Xianlin Yi

**Affiliations:** ^1^ Department of Urology, The Second Affiliated Hospital of Guangxi Medical University, Nanning, Guangxi, China; ^2^ Department of Urology, Liuzhou Worker’s Hospital, Liuzhou, Guangxi, China; ^3^ Department of Urology, Guangxi Medical University Cancer Hospital, Nanning, Guangxi, China; ^4^ Department of Graduate School, Guangxi Medical University, Nanning, Guangxi, China; ^5^ Department of Radiology, Guangxi Medical University Cancer Hospital, Nanning, Guangxi, China; ^6^ Department of Urology, Wuming Hospital of Guangxi Medical University, Nanning, Guangxi, China; ^7^ Department of Urology, Maternal and Child Health Hospital of Hubei Province, Tongji Medical College, Huazhong University of Science and Technology, Wuhan, Hubei, China

**Keywords:** lactate dehydrogenase-to-serum albumin ratio, platelet-to-lymphocyte ratio, fibrinogen-to-albumin ratio, high-grade urothelial carcinoma, prognosis

## Abstract

**Objective:**

We evaluated the prognostic significance of the Lactate Dehydrogenase-to-Serum Albumin Ratio (LAR), Fibrinogen-to-Albumin Ratio (FAR), and Platelet-to-Lymphocyte Ratio (PLR) in patients with high-grade urothelial carcinoma (HGUC) of the bladder who underwent radical cystectomy (RC). These markers have been reported to be associated with the prognosis of various cancers.

**Methods:**

A retrospective analysis was conducted on HGUC patients who underwent RC at Guangxi Medical University Cancer Hospital between January 2013 and June 2021. Optimal cutoff values for LAR, FAR, and PLR were established. Kaplan-Meier survival analysis was used to evaluate survival outcomes, while univariate and multivariable Cox regression analyses identified independent prognostic factors. A nomogram was developed to predict survival, with validation through time-dependent receiver operating characteristic (ROC) curves, calibration plots, and decision curve analysis (DCA).

**Results:**

A total of 180 patients were included, with a follow-up period ranging from 2 to 127 months (49.28 ± 37.87 months). The optimal cutoff values for LAR, PLR, and FAR were 4.46, 139.68, and 0.13, respectively. Multivariable Cox regression identified tumor stage, LAR, PLR, and FAR as independent prognostic factors. Specifically, Stage III (HR = 25.44, 95% CI: 5.20–124.35, p < 0.001) and Stage IV (HR = 11.28, 95% CI: 3.18–40.05, p < 0.001) were independent risk factors for poor survival. A low PLR (HR = 0.45, 95% CI: 0.27–0.76, p = 0.003), low FAR (HR = 0.51, 95% CI: 0.29–0.89, p = 0.018), and low LAR (HR = 0.39, 95% CI: 0.23–0.67, p < 0.001) were independently associated with improved survival. The nomogram demonstrated high accuracy in predicting 1-, 3-, and 5-year overall survival (OS), with area under the curve (AUC) values of 0.866, 0.84, and 0.831, respectively. Further validation confirmed the model’s stability and clinical applicability.

**Conclusion:**

LAR, PLR, and FAR are promising prognostic factors for HGUC of the bladder following RC, showing substantial potential for prognostic evaluation.

## 1 Introduction

Bladder cancer represents a significant global public health challenge and is the second most common malignancy of the urinary system. According to 2022 data, bladder cancer ranks ninth in global incidence and thirteenth in cancer-related mortality (1). Among bladder cancers, urothelial carcinoma is the most prevalent histological subtype, which can be classified into low-grade and high-grade urothelial carcinoma based on pathological grading (2). HGUC is associated with a higher recurrence rate and increased mortality compared to low-grade urothelial carcinoma (3, 4). Despite significant advances in bladder cancer treatment, including surgery, chemotherapy, and immunotherapy, RC remains a common treatment option for patients with HGUC that is clinically staged as high T without distant metastasis (5, 6). However, HGUC is highly malignant, strongly correlating with tumor progression and cancer death rates (3). Therefore, there is an urgent need for effective prognostic factor to predict clinical outcomes more accurately and to guide personalized treatment strategies.

In recent years, there has been increasing interest in hematological indices associated with systemic inflammation and immune status. Studies have shown that indicators such as the LAR, PLR, and FAR are closely linked to the prognosis of various cancers (7–9). Inflammation and immune responses can promote tumor proliferation, invasion, and other mechanisms, accelerating cancer progression (10). Although some studies suggest an association between serum inflammatory markers and bladder cancer prognosis, their clinical significance and prognostic value in HGUC of the bladder have yet to be fully validated. Thus, further reliable evidence is required to explore the clinical applicability of these factors (11–13).

This study aims to explore the potential of hematological indicators, including LAR, PLR, and FAR, as prognostic tools for HGUC of the bladder by analyzing their correlation with OS following RC. The ultimate goal is to construct a nomogram model that can provide more accurate predictions of OS for bladder cancer patients following RC.

## 2 Patients and methods

### 2.1 Study subjects

This retrospective study analyzed the clinical data of 180 patients with HGUC of the bladder who underwent RC at the Department of Urology, Guangxi Medical University Cancer Hospital, between January 2013 and June 2021. Tumor histological type, grade, and stage were determined according to the 2016 WHO classification and the 8th edition of the American Joint Committee on Cancer (AJCC) staging manual (2, 6). The inclusion criteria were: (I) histologically or clinically diagnosed HGUC≥T1; (II) received RC; (III) complete routine preoperative blood test results within one week prior to surgery; (IV) aged 18 years or older; (V) complete clinical data and follow-up information. The exclusion criteria were: (I) concurrent malignancies; (II) comorbidities affecting survival, such as multi-organ failure, liver disease, autoimmune diseases, hematological disorders, and infectious diseases; (III) history of preoperative antitumor therapy, including radiotherapy, chemotherapy, or immunotherapy; (IV) incomplete clinical or pathological data; (V) loss to follow-up or incomplete follow-up data.

The surgical approaches include open and laparoscopic radical cystectomy, both accompanied by urinary diversion. Radical cystectomy is employed for patients with locally resectable muscle-invasive bladder cancer (stages T2-T4a) or high-risk T1, particularly in cases of multifocality or recurrence. For selected patients with advanced-stage disease, palliative cystectomy is performed to alleviate symptoms and improve quality of life.

### 2.2 Study methods

Clinical data, including patient age, sex, surgical method, pathological results, TNM stage, grade, muscularis infiltration, lymph node metastasis, and distant metastasis, were collected. Preoperative blood test data were also collected, and several indicators were calculated using the following formulas: NLR = neutrophil count/lymphocyte count; LAR = lactate dehydrogenase/serum albumin; PLR = platelet count/lymphocyte count; SII = (neutrophil count × platelet count)/lymphocyte count; MLR = monocyte count/lymphocyte count; FAR = fibrinogen/albumin; PIV = (neutrophil count × platelet count × monocyte count)/lymphocyte count. The optimal cutoff values for NLR, PLR, SII, MLR, FAR, LAR, and PIV were determined using Kaplan-Meier survival analysis with the survminer R package, and patients were divided into high and low groups. Kaplan-Meier survival analysis and log-rank tests were performed using the Survival R package.

### 2.3 Follow-up

Patients were advised to undergo chest and abdominal CT scans every 3–6 months during the first two years post-surgery, followed by annual scans thereafter. Follow-up was conducted via telephone or outpatient visits to monitor patients’ general condition. The follow-up period ended in June 2024. OS was defined as the time from surgery to death.

### 2.4 Statistical analysis

Data were analyzed using R software (version 4.4.1), IBM SPSS Statistics (version 25), and Zstats 1.0 (www.zstats.net). Continuous variables were expressed as mean ± standard deviation (SD), and categorical variables were presented as percentages. Fisher’s exact test or the chi-square test was used for categorical variables, and t-tests or nonparametric tests were applied to continuous variables based on their distribution. In univariate Cox regression analysis, variables with a p-value < 0.05 were included in the multivariable Cox analysis, and stepwise regression was used to determine the final variables for constructing the nomogram. The accuracy of the nomogram was evaluated using ROC curves and calibration plots. DCA was used to assess the clinical utility of the nomogram, with curves above the “None” and “All” lines indicating a net benefit, suggesting the model’s applicability in clinical practice. Statistical significance was defined as P < 0.05.

This study was approved by the Ethics Committee of Guangxi Medical University Cancer Hospital (No. KY2020069), and all procedures were performed in accordance with the Declaration of Helsinki. The requirement for written informed consent was waived due to the retrospective nature of the study.

## 3 Results

### 3.1 Patient characteristics

**Figure 1:**
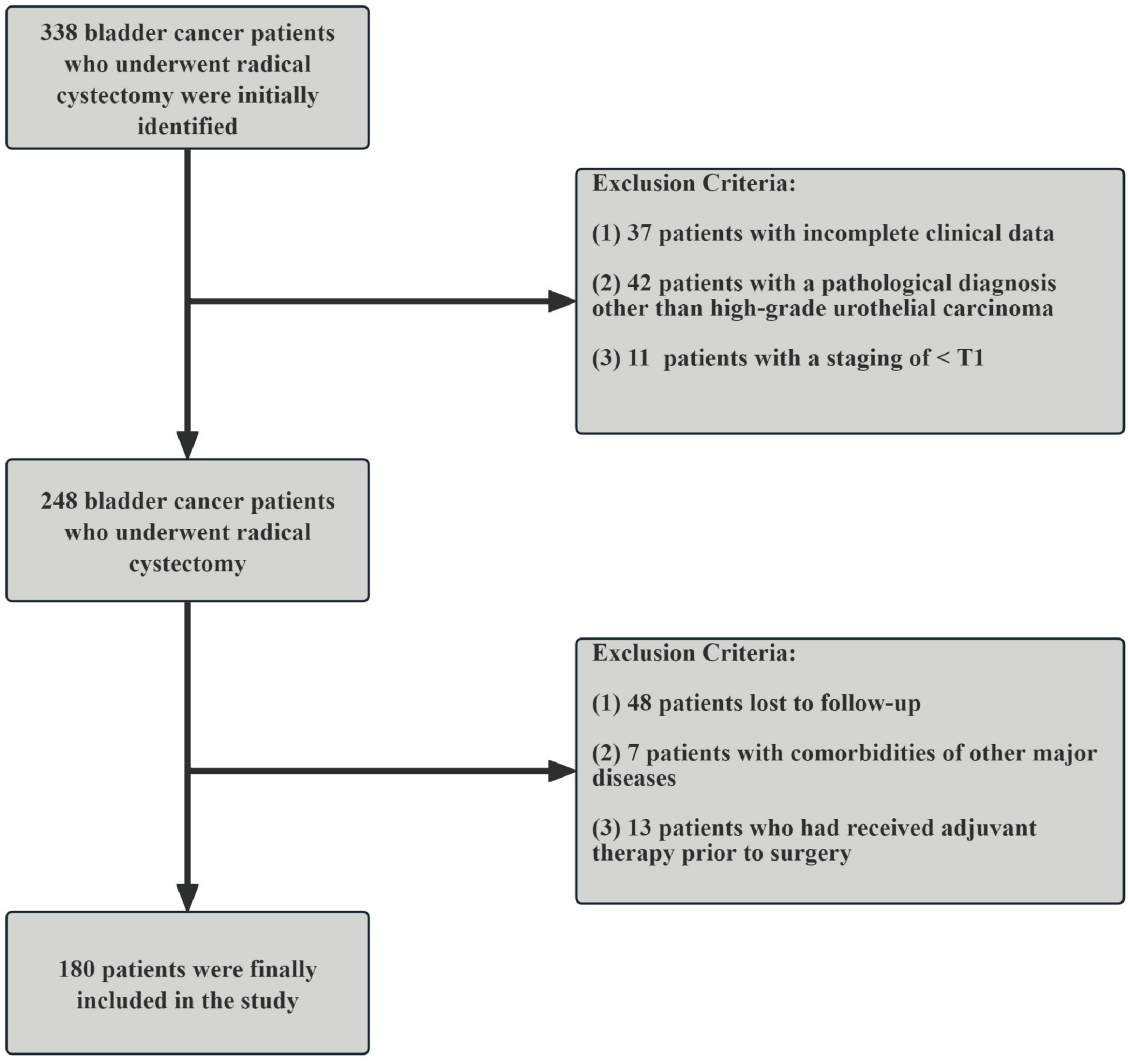
Flowchart of study patients.

**Figure 2:**
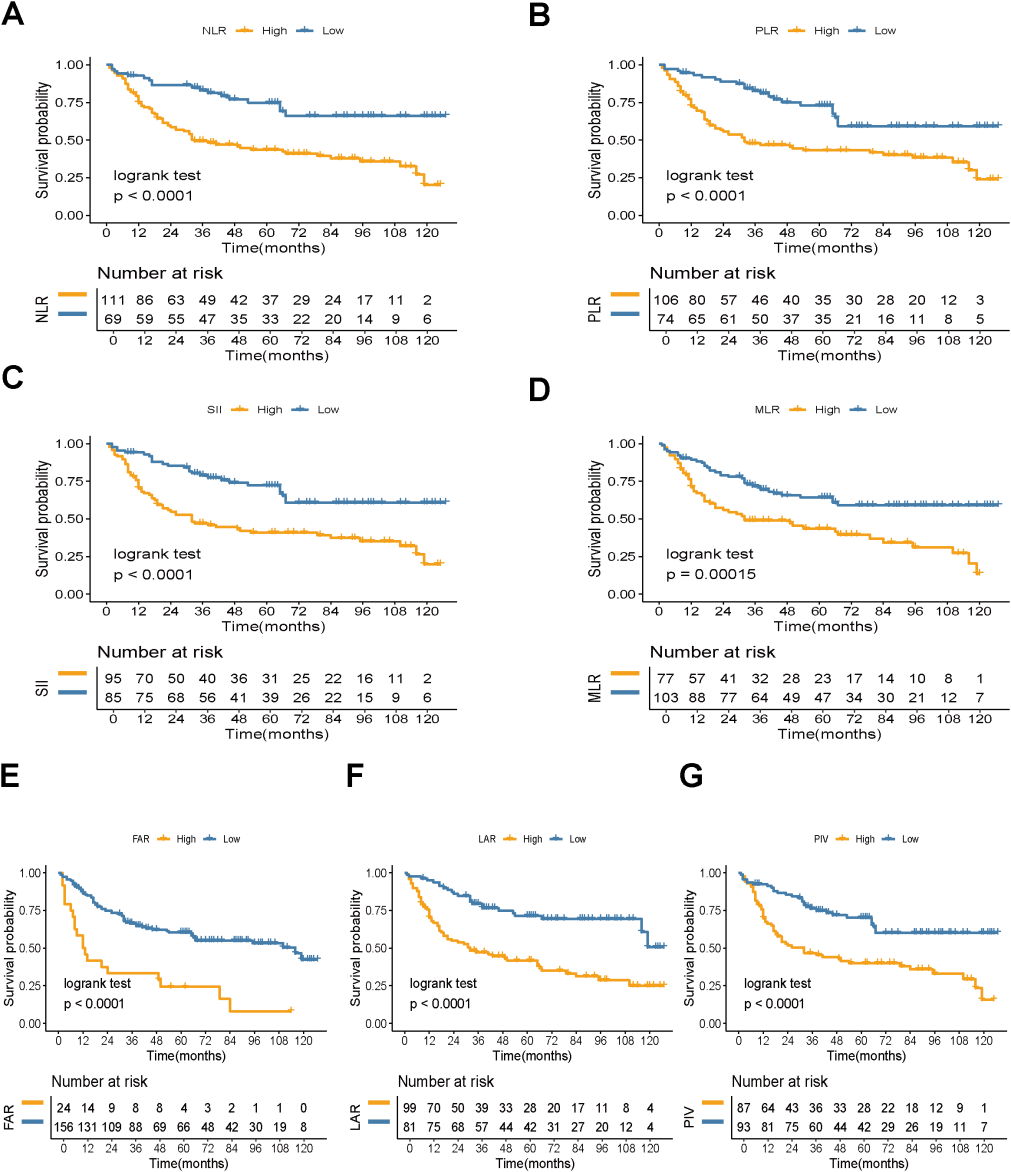
Kaplan-Meier estimate of OS. Kaplan-Meier survival curves for OS based on **(A)** NLR, **(B)** PLR, **(C)** SII, **(D)** MLR, **(E)** FAR, **(F)** LAR, and **(G)** PIV levels (log-rank test, P < 0.05). NLR, neutrophil-to-lymphocyte ratio; PLR, platelet-to-lymphocyte ratio; SII, systemic immune-inflammation index; MLR, monocyte-to-lymphocyte ratio; FAR, fibrinogen-to-albumin ratio; LAR, lactate dehydrogenase-to-albumin ratio; PIV, pan-immune-inflammation value. OS, overall survival.

A total of 180 patients who underwent RC were included in this study (**Figure 1**), with follow-up periods ranging from 2 to 127 months (49.28 ± 37.87 months). Among these, 161 were male (89.44%) and 19 were female (10.56%). The mean age was 66.32 ± 11.50 years. The patients were categorized into two groups based on survival status: 94 patients were alive (52.22%) and 86 patients had died (47.78%). Regarding clinical and pathological features, 82 patients (45.56%) were in stage T1, while 98 patients (54.44%) had tumors staged >T1. N0 status was observed in 147 patients (81.67%), while 33 patients (18.33%) had ≥N0 status. M0 status was found in 164 patients (91.11%), and 16 patients (8.89%) had M1 status. Muscle invasion was present in 102 patients (56.67%), while 78 patients (43.33%) had non-muscle-invasive bladder cancer. The optimal cutoff values for NLR, PLR, SII, MLR, FAR, LAR, and PIV were 2.16, 139.68, 648.05, 0.29, 0.13, 4.46, and 333.27, respectively. Based on these cutoff values, patients were classified into high and low groups. Kaplan-Meier analysis indicated that higher preoperative NLR, PLR, SII, MLR, FAR, LAR, and PIV were associated with poorer OS (log-rank P < 0.001, **Figure 2**). Detailed baseline clinical characteristics of the study population are presented in **Table 1**.

**Table 1:** Baseline characteristics of the study population.

Variables	Total (n = 180)	Group		Statistic	*P*
		Survival group (n = 94)	Death group (n = 86)		
Age, Mean ± SD	66.32 ± 11.50	63.67 ± 10.87	69.22 ± 11.54	t=-3.32	**0.001**
Gender, n (%)				χ²=2.01	0.156
Male	161 (89.44)	87 (92.55)	74 (86.05)		
Female	19 (10.56)	7 (7.45)	12 (13.95)		
T, n (%)				χ²=31.53	**<.001**
T1	82 (45.56)	55 (58.51)	27 (31.40)		
T2	50 (27.78)	30 (31.91)	20 (23.26)		
T3	26 (14.44)	7 (7.45)	19 (22.09)		
T4	22 (12.22)	2 (2.13)	20 (23.26)		
N, n (%)				–	**<.001**
N0	147 (81.67)	87 (92.55)	60 (69.77)		
N1	18 (10.00)	7 (7.45)	11 (12.79)		
N2	13 (7.22)	0 (0.00)	13 (15.12)		
N3	2 (1.11)	0 (0.00)	2 (2.33)		
M, n (%)				χ²=14.88	**<.001**
M0	164 (91.11)	93 (98.94)	71 (82.56)		
M1	16 (8.89)	1 (1.06)	15 (17.44)		
Muscle invasive, n (%)				χ²=11.51	**<.001**
Yes	102 (56.67)	42 (44.68)	60 (69.77)		
No	78 (43.33)	52 (55.32)	26 (30.23)		
Lymph Node Metastasis, n (%)				χ²=15.57	**<.001**
Yes	33 (18.33)	7 (7.45)	26 (30.23)		
No	147 (81.67)	87 (92.55)	60 (69.77)		
Distant Metastasis, n (%)				χ²=16.16	**<.001**
Yes	17 (9.44)	1 (1.06)	16 (18.60)		
No	163 (90.56)	93 (98.94)	70 (81.40)		
Stage, n (%)				–	**<.001**
I	78 (43.33)	54 (57.45)	24 (27.91)		
II	39 (21.67)	25 (26.60)	14 (16.28)		
IIIA	37 (20.56)	14 (14.89)	23 (26.74)		
IIIB	8 (4.44)	0 (0.00)	8 (9.30)		
IV	18 (10.00)	1 (1.06)	17 (19.77)		
NLR, n (%)				χ²=21.10	**<.001**
High	111 (61.67)	43 (45.74)	68 (79.07)		
Low	69 (38.33)	51 (54.26)	18 (20.93)		
PLR, n (%)				χ²=16.41	**<.001**
High	106 (58.89)	42 (44.68)	64 (74.42)		
Low	74 (41.11)	52 (55.32)	22 (25.58)		
SII, n (%)				χ²=19.07	**<.001**
High	95 (52.78)	35 (37.23)	60 (69.77)		
Low	85 (47.22)	59 (62.77)	26 (30.23)		
MLR, n (%)				χ²=13.56	**<.001**
High	77 (42.78)	28 (29.79)	49 (56.98)		
Low	103 (57.22)	66 (70.21)	37 (43.02)		
FAR, n (%)				χ²=14.03	**<.001**
High	24 (13.33)	4 (4.26)	20 (23.26)		
Low	156 (86.67)	90 (95.74)	66 (76.74)		
LAR, n (%)				χ²=19.44	**<.001**
High	99 (55.00)	37 (39.36)	62 (72.09)		
Low	81 (45.00)	57 (60.64)	24 (27.91)		
PIV, n (%)				χ²=18.57	**<.001**
High	87 (48.33)	31 (32.98)	56 (65.12)		
Low	93 (51.67)	63 (67.02)	30 (34.88)		

t, t-test; χ², Chi-square test; -, Fisher exact; SD, standard deviation.

NLR, neutrophil-lymphocyte ratio; PLR, platelet-lymphocyte ratio; SII, systemic immune-inflammation index; MLR, Monocyte-to-Lymphocyte Ratio; FAR, fibrinogen–albumin ratio; LAR, lactate-albumin; PIV, pan-immune inflammation value. Bold values indicate statistically significant differences (P<0.05).

### 3.2 Univariate and multivariable Cox regression analysis

Univariate Cox regression analysis identified age, tumor TNM stage, muscle invasion, lymph node metastasis, distant metastasis, NLR, PLR, SII, MLR, FAR, LAR, and PIV as significant factors associated with overall survival (p < 0.05) (**Table 2**). Variables with p < 0.05 in the univariate analysis were subsequently included in the multivariable Cox regression model. The results indicated that tumor stage, PLR, FAR, and LAR were independent prognostic factors for survival following RC. Stage III (HR = 25.44, 95% CI: 5.20–124.35, p < 0.001) and Stage IV (HR = 11.28, 95% CI: 3.18–40.05, p < 0.001) were independent risk factors for poor survival. Low PLR was associated with improved survival (HR = 0.45, 95% CI: 0.27–0.76, p = 0.003), low FAR was associated with better survival (HR = 0.51, 95% CI: 0.29–0.89, p = 0.018), and low LAR was an independent predictor of better survival (HR = 0.39, 95% CI: 0.23–0.67, p < 0.001).

**Table 2:** Univariate and multivariable cox regression analysis for overall survival.

Variables	Univariate Analysis					Multivariable Analysis				
	β	S.E	Z	*P*	HR (95%CI)	β	S.E	Z	*P*	HR (95%CI)
Age	0.03	0.01	2.72	**0.007**	1.03 (1.01 ~ 1.05)					
Gender										
Male					1.00 (Reference)					
Female	0.59	0.31	1.89	0.059	1.80 (0.98 ~ 3.32)					
T										
T1					1.00 (Reference)					1.00 (Reference)
T2	0.08	0.30	0.28	0.780	1.09 (0.61 ~ 1.95)	-0.68	0.76	-0.90	0.369	0.50 (0.11 ~ 2.24)
T3	1.19	0.30	3.96	**<.001**	3.30 (1.83 ~ 5.96)	-1.02	0.70	-1.46	0.144	0.36 (0.09 ~ 1.42)
T4	1.95	0.30	6.43	**<.001**	7.02 (3.87 ~ 12.71)	0.18	0.66	0.27	0.787	1.20 (0.33 ~ 4.37)
Muscle invasive										
Yes					1.00 (Reference)					
No	-0.78	0.24	-3.29	**0.001**	0.46 (0.29 ~ 0.73)					
Lymph Node Metastasis										
Yes					1.00 (Reference)					
No	-1.17	0.24	-4.93	**<.001**	0.31 (0.19 ~ 0.49)					
Distant Metastasis										
Yes					1.00 (Reference)					
No	-1.53	0.29	-5.30	**<.001**	0.22 (0.12 ~ 0.38)					
Stage										
I					1.00 (Reference)					1.00 (Reference)
II	0.04	0.34	0.13	0.896	1.05 (0.54 ~ 2.03)	0.91	0.83	1.10	0.273	2.48 (0.49 ~ 12.58)
IIIA	0.93	0.29	3.17	**0.002**	2.54 (1.43 ~ 4.52)	1.11	0.70	1.59	0.112	3.03 (0.77 ~ 11.85)
IIIB	2.56	0.43	5.97	**<.001**	12.88 (5.57 ~ 29.80)	3.24	0.81	4.00	**<.001**	25.44 (5.20 ~ 124.35)
IV	2.03	0.33	6.17	**<.001**	7.63 (4.00 ~ 14.55)	2.42	0.65	3.75	**<.001**	11.28 (3.18 ~ 40.05)
NLR										
High					1.00 (Reference)					
Low	-1.07	0.27	-4.02	**<.001**	0.34 (0.20 ~ 0.58)					
PLR										
High					1.00 (Reference)					1.00 (Reference)
Low	-0.93	0.25	-3.75	**<.001**	0.39 (0.24 ~ 0.64)	-0.79	0.26	-3.00	**0.003**	0.45 (0.27 ~ 0.76)
SII										
High					1.00 (Reference)					
Low	-0.96	0.24	-4.09	**<.001**	0.38 (0.24 ~ 0.61)					
MLR										
High					1.00 (Reference)					
Low	-0.80	0.22	-3.67	**<.001**	0.45 (0.29 ~ 0.69)					
FAR										
High					1.00 (Reference)					1.00 (Reference)
Low	-1.18	0.26	-4.59	**<.001**	0.31 (0.18 ~ 0.51)	-0.67	0.28	-2.36	**0.018**	0.51 (0.29 ~ 0.89)
LAR										
High					1.00 (Reference)					1.00 (Reference)
Low	-1.12	0.24	-4.62	**<.001**	0.33 (0.20 ~ 0.53)	-0.93	0.27	-3.44	**<.001**	0.39 (0.23 ~ 0.67)
PIV										
High					1.00 (Reference)					
Low	-0.94	0.23	-4.16	**<.001**	0.39 (0.25 ~ 0.61)					

HR, Hazards Ratio; CI, Confidence Interval.

*p *< 0.05, The difference is statistically significant.

OS, overall survival; NLR, neutrophil-to-lymphocyte ratio; PLR, platelet-to-lymphocyte ratio; SII, systemic immune-inflammation index; MLR, monocyte-to-lymphocyte ratio; FAR, fibrinogen-to-albumin ratio; LAR, lactate dehydrogenase-to-albumin ratio; PIV, pan-immune-inflammation value. OS, overall survival. Bold values indicate statistically significant differences (P<0.05).

### 3.3 Construction and evaluation of the nomogram

#### 3.3.1 Nomogram construction

**Figure 3:**
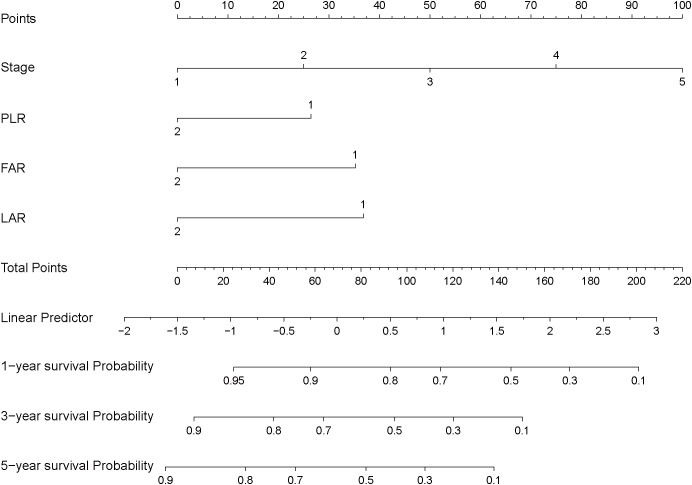
Nomogram for predicting postoperative survival in high-grade urothelial carcinoma of the bladder.

Based on the results of the multivariable Cox regression analysis, we constructed a nomogram to predict the 1-year, 3-year, and 5-year survival probabilities for patients with HGUC of the bladder following RC. The nomogram includes independent risk factors such as tumor stage, PLR, FAR, and LAR (**Figure 3**).

#### 3.3.2 Time-dependent ROC

**Figure 4:**
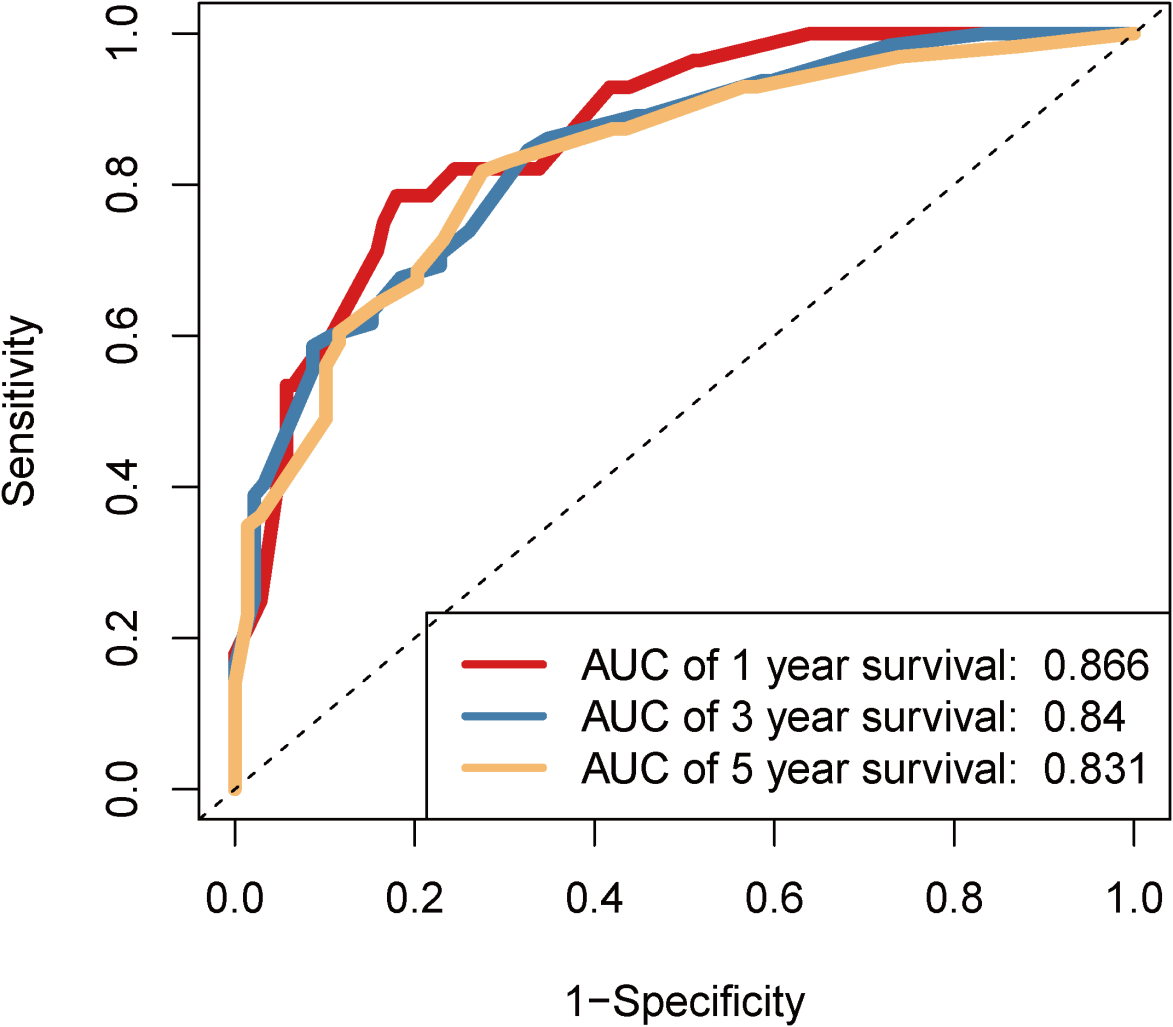
Time-dependent ROC Curve of the Nomogram.

We performed time-dependent ROC curve analysis (**Figure 4**) to evaluate the predictive performance of the nomogram for OS in patients with HGUC following RC. The AUC for 1-year survival was 0.866, indicating excellent predictive accuracy. For 3-year and 5-year survival predictions, the AUCs were 0.84 and 0.831, respectively, demonstrating that the model maintains stable and reliable predictive performance over longer follow-up periods. These results validate the reliability and stability of the nomogram in clinical settings, underscoring its practical value.

#### 3.3.3 Calibration plot

**Figure 5:**
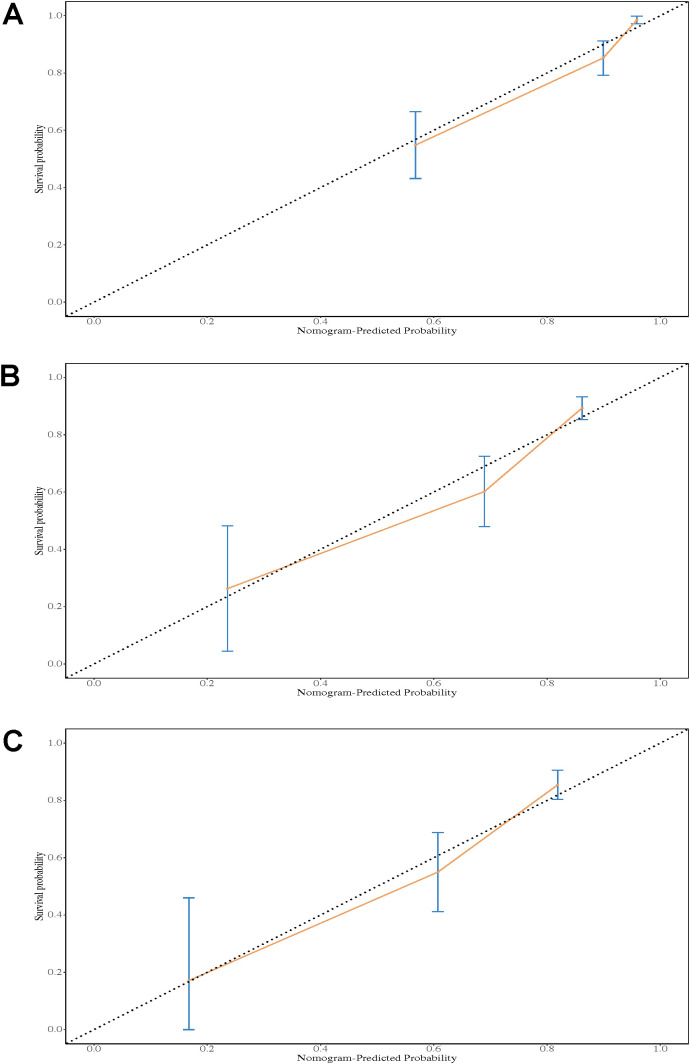
Calibration plot of the nomogram for 1-year OS **(A)**, 3-year OS **(B)**, and 5-year OS **(C)**.

The calibration plot showed that the nomogram provided accurate and consistent predictions for the 1-year, 3-year, and 5-year overall survival probabilities in patients with HGUC after RC. The 1-year survival prediction closely aligned with the observed outcomes, with the curve near the 45-degree reference line and a narrow confidence interval, reflecting high prediction accuracy (**Figure 5A**). The 3-year survival prediction was generally consistent, although there was slight overestimation in the low-probability range (**Figure 5B**). The 5-year survival prediction performed well in the medium-probability range but showed some variation in extreme values (**Figure 5C**).

#### 3.3.4 DCA

**Figure 6:**
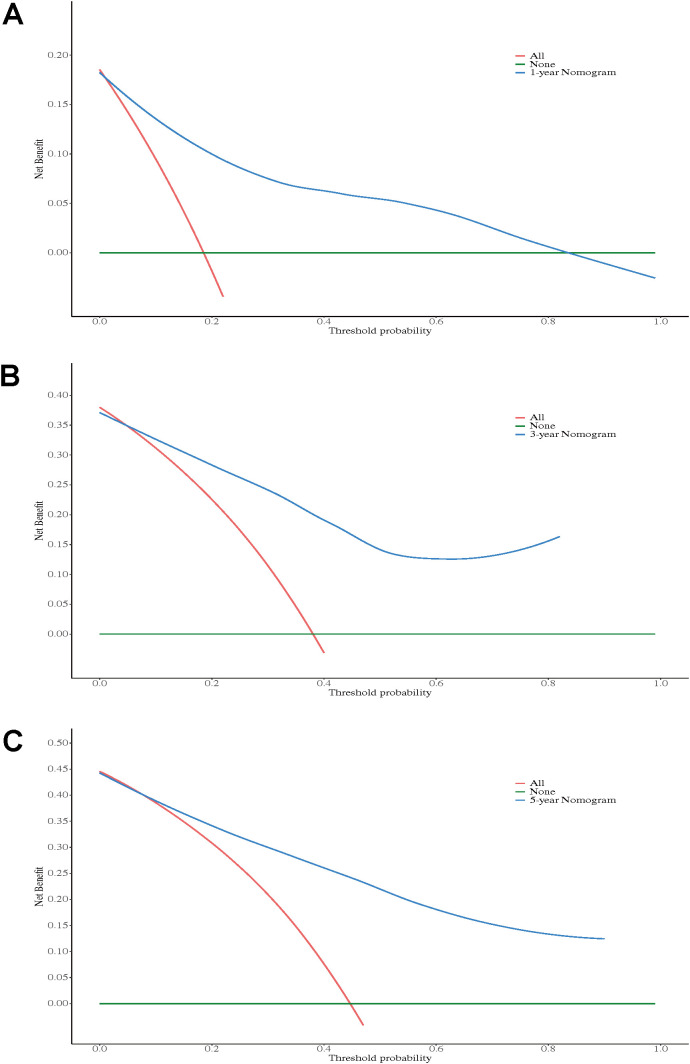
Decision curve analysis of the nomogram for 1-year OS **(A)**, 3-year OS **(B)**, and 5-year OS **(C)**.

DCA demonstrated that the postoperative nomogram provided significant clinical value in predicting outcomes at 1-, 3-, and 5-year following RC. The 1-year nomogram (**Figure 6A**) showed net benefits in the threshold probability range of 0.05 to 0.30, the 3-year nomogram (**Figure 6B**) in the range of 0.05 to 0.40, and the 5-year nomogram (**Figure 6C**) in the range of 0.05 to 0.45, all outperforming the “treat all” and “treat none” strategies. These findings suggest that the postoperative nomogram can effectively support clinical decision-making.

Overall, the nomogram demonstrates high practical value in predicting overall survival after RC.

## 4 Discussion

HGUC of the bladder is a highly aggressive malignancy, characterized by high recurrence rates and poor prognosis (2, 14). It remains one of the most challenging urological cancers to treat. This study aimed to explore the relationship between preoperative blood biomarkers and OS in patients with HGUC undergoing RC, with the goal of evaluating the prognostic potential of these markers. Our findings suggest that tumor stage, LAR, FAR, and PLR are independent prognostic factors for patient survival. Based on these variables, we developed and validated a nomogram, providing a significant prognostic tool for clinical assessment in patients after RC for HGUC.

Inflammatory responses are crucial in tumor initiation, progression, and metastasis, and are closely associated with a variety of cancers (15). Bladder urothelial carcinoma is strongly linked to systemic inflammation, with chronic inflammatory states being carcinogenic. Inflammatory macrophages and lymphocytes increase the production of reactive oxygen species (ROS) and reactive nitrogen species (RNS), which promote bladder cancer progression (16). Additionally, studies have shown that bladder tumors are correlated with inflammatory markers in the blood, such as CRP, CD3+, and CD8+ cells, all of which are significantly associated with survival outcomes (13). The role of immune-inflammatory responses in the progression of bladder cancer has been extensively investigated, and research on blood biomarkers associated with bladder cancer progression has gradually increased. Felice Crocetto et al. have proposed that current blood-based immune-inflammatory biomarkers are of significant prognostic value in the progression of bladder cancer (17, 18). Our results further suggest that preoperative LAR, FAR, and PLR are strongly associated with prognosis in bladder cancer patients, underscoring the pivotal role of inflammation in the prognosis of bladder cancer.

LAR, the ratio of lactate dehydrogenase (LDH) to serum albumin, has emerged as a reliable prognostic marker. LDH is a key enzyme in aerobic glycolysis, converting pyruvate to lactate and creating a mildly hypoxic environment that contributes to tumor hypoxia, angiogenesis, and poor prognosis (19). Elevated LDH levels are associated with poor prognosis in bladder cancer (20). Serum albumin, an important biomarker for assessing nutritional status, is frequently low in advanced malignancies. Previous studies have shown that low serum albumin levels correlate with poor cancer prognosis (21). When combined, LAR, incorporating both LDH and albumin, provides a more reliable indicator than either marker alone. Several studies have demonstrated the prognostic value of preoperative LAR in cancers such as nasopharyngeal carcinoma, breast cancer, and colorectal cancer (7, 22, 23). Our study further found that a preoperative LAR >4.46(HR = 0.39, 95% CI: 0.23–0.67, p < 0.001) is associated with poor OS in HGUC patients following RC.

PLR, a marker reflecting the ratio of platelets to lymphocytes, is also indicative of inflammation and immune response, and has demonstrated prognostic value in various cancers (24–26). Platelets play a critical role in tumor growth, metastasis, angiogenesis, and immune-inflammatory responses (27), while lymphocytes are integral to tumor immune surveillance (28). Furthermore, lymphocytes are crucial for immune defense, promoting the apoptosis of cytotoxic cells and inhibiting the proliferation and migration of tumor cells. A reduction in lymphocytes can impair anti-tumor immunity (29). We observed that lower PLR(HR = 0.45, 95% CI: 0.27–0.76, p = 0.003) values were associated with better prognosis, consistent with findings in other cancer types. An elevated PLR likely reflects an immunosuppressive state, holding significant prognostic value in bladder cancer.

FAR, the ratio of fibrinogen to albumin, has gained attention as a biomarker that reflects both systemic inflammation and nutritional status. However, its mechanisms in HGUC prognosis remain incompletely understood. Previous studies have shown that fibrinogen induces the expression of intercellular adhesion molecule-1 (ICAM-1), promoting tumor cell migration, angiogenesis, and metastasis in various cancers, including gallbladder cancer (30). Elevated plasma fibrinogen levels have also been closely linked to poor prognosis in non-muscle-invasive bladder cancer (31). Decreased serum albumin levels not only impair immune function but may also promote pro-inflammatory factors associated with cancer progression, increasing cancer risk (32). Low serum albumin has been identified as an independent predictor of poor long-term prognosis in bladder cancer, with an increased risk of postoperative complications and mortality.

A meta-analysis has demonstrated that higher FAR is significantly associated with OS and disease-free survival DFS in breast cancer patients. Similarly, a single-center study involving patients who underwent transurethral resection of bladder tumors found that elevated preoperative FAR levels serve as a predictive indicator for advanced bladder cancer (18). In line with this, our study identified high FAR as a risk factor for poor OS in HGUC patients undergoing RC, further supporting FAR as a potential prognostic marker in RC for HGUC patients. Preoperative lower FAR (HR = 0.51, 95% CI: 0.29–0.89, p = 0.018) may also serve as an important reference factor for clinicians when assessing patient prognosis.

Through univariate and multivariable Cox regression analyses, we confirmed that tumor stage, PLR, FAR, and LAR are independent prognostic factors. Tumor stage, particularly Stage III and IV, was identified as an independent risk factor for poor survival. Lower values of PLR, FAR, and LAR were associated with improved survival outcomes. In the multivariable analysis, the T stage did not achieve statistical significance, which may be attributable to other confounding factors or limitations in sample size. However, the P value for the Stage in the multivariable analysis was statistically significant. We propose that, although the T stage plays a crucial role in treatment decision-making for bladder cancer, incorporating the Stage based on the TNM classification into the analysis may provide a more comprehensive prognostic assessment. Based on these results, we developed a nomogram model that could serve as a personalized guide for treatment decisions.

Our study has several limitations. First, due to its retrospective design, selection bias may have been introduced, leading to an uneven distribution of certain characteristics (e.g., age, stage, and gender) within the study sample. Additionally, postoperative adjuvant therapy, as a potential confounding factor, may have impacted the accuracy of the findings. Second, the relatively small sample size and the use of data from a single center may have resulted in a slight overestimation of the predictive probabilities, and also limit the generalizability of the results. Therefore, future studies should involve larger, multicenter, prospective cohorts to validate these findings and enhance their external validity. Finally, this study did not investigate the specific molecular mechanisms by which LAR, FAR, and PLR influence prognosis in HGUC of the bladder. Future research should focus on elucidating the molecular roles of these factors in bladder cancer, which could offer more precise clinical guidance.

## 5 Conclusions

This study identifies LAR, FAR and PLR as independent prognostic factors for patients with HGUC of the bladder undergoing RC. The nomogram developed in this study demonstrates high predictive accuracy and considerable clinical utility. However, further validation through large-scale, multicenter studies is needed.

## Data Availability

The original contributions presented in the study are included in the article/supplementary material. Further inquiries can be directed to the corresponding authors.
